# Radiomic model with SHAP-based interpretability for predicting invasiveness of pure ground-glass nodules: a retrospective study based on high-resolution computed tomography (HRCT) volumetric datasets

**DOI:** 10.1186/s13019-026-04560-5

**Published:** 2026-07-18

**Authors:** Hui Sheng, Rui Wang, Guowei Zhang, Ning Dong, Yunpeng Zhou, Ping Wang, Kexin Li, Guojie Bai

**Affiliations:** 1 Department of Radiology, Shengli Oilfield Central Hospital, Dongying, 257000 China; 2 Department of Respiratory Medicine, Dongying Hospital of Traditional Chinese Medicine, Dongying, 257000 Shandong China; 3 Department of Radiology, Yantaishan Hospital, Yantai, 264000 Shandong China; 4 Department of Radiology, Yantai Yuhuangding Hospital, Tianjin, 264000 Shandong China; 5 Department of Radiology, Tianjin Beichen Traditional Chinese Medicine Hospital, Tianjin, 300400 China

**Keywords:** Pure ground-glass nodules, High-resolution computed tomography, Radiomics, Interpretable artificial intelligence

## Abstract

**Background:**

Radiomics holds promise for lung cancer diagnosis. This study developed an interpretable radiomics–clinical model to predict the invasiveness of pure ground-glass nodules (pGGNs) on high-resolution computed tomography (HRCT). To address the model’s “black box” nature, we applied the SHapley Additive exPlanations (SHAP) framework.

**Methods:**

We retrospectively analyzed 235 surgically resected, histopathologically confirmed pGGNs, classified as non-invasive (AAH/AIS/MIA) or invasive (IAC) according to the 2015 WHO classification of lung tumors. We developed three prediction models: clinical, radiomic, and combined. Feature selection for the radiomic and combined models employed LASSO regression. Model performance was assessed using AUC, calibration curves, and decision curve analysis (DCA). Additionally, SHAP was used to quantify feature importance and to generate individualized explanations.

**Results:**

Two clinico-radiological features (mean CT value, VolumePercent₋₃₀₀) and eight radiomic features were retained. The combined model yielded AUCs of 0.923 (training) and 0.832 (testing), outperforming the clinical model (0.799/0.733) and the radiomic model (0.917/0.827). Decision curve analysis (DCA) confirmed the superior clinical utility of the combined model. SHAP analysis ranked log_sigma_2_0mm_3D_firstorder_Range as the single most important predictive feature.

**Conclusions:**

The SHAP-augmented radiomics–clinical model offers an accurate and interpretable preoperative assessment of pGGN invasiveness. This tool can help clinicians choose the optimal surgical strategy and support individualized decision-making.

**Supplementary Information:**

The online version contains supplementary material available at https://doi.org/10.1186/s13019-026-04560-5.

## Background

The widespread adoption of high-resolution computed tomography (HRCT) has increased detection of pulmonary nodules—specifically pure ground-glass nodules (pGGNs)—during routine lung cancer screening [1]. Radiologically, pGGNs appear as faint, poorly circumscribed areas of increased lung attenuation that do not obscure bronchial or vascular margins [2]. These imaging findings reflect a histopathologic continuum of early pulmonary adenocarcinoma, ranging from atypical adenomatous hyperplasia (AAH) through adenocarcinoma in situ (AIS) and minimally invasive adenocarcinoma (MIA) to invasive adenocarcinoma (IAC) [3].

Although pGGNs often appear indolent on imaging, a substantial fraction contain invasive histology. Studies report that approximately 40% to 55% of pGGNs are histologically invasive [4]. Moreover, lesions that are initially stable on imaging may progress over time, underscoring the need for accurate preoperative assessment [5]. Pre-invasive lesions can frequently be observed or treated with limited resection, whereas invasive lesions typically require lobectomy with lymph node dissection to achieve optimal outcomes [6].

CT-based evaluation of pGGN invasiveness depends on imaging features such as size, mean CT attenuation (Hounsfield units, HU), margins, and spiculation. To increase diagnostic accuracy, HRCT has been introduced. HRCT acquires thin slices (≤ 1 mm), employs a high matrix resolution, and limits the field of view to boost spatial resolution and reveal fine internal details, including air bronchograms and vessel convergence. Compared with standard CT, HRCT enables a more precise morphological evaluation of subtle lesions, particularly in early-stage disease [7].

Despite advances in imaging technology, accurate diagnosis remains challenging. Radiological interpretation still depends heavily on individual radiologists’ expertise, and subjective assessments often produce interobserver variability [8, 9]. Clinically, using morphology alone to distinguish pre-invasive from invasive lesions is frequently inconsistent and unreliable [10, 11].

Radiomic methods have emerged as a promising strategy. By extracting quantitative features from medical images and applying machine learning, they enable objective, reproducible profiling of lesion heterogeneity. Prior studies have demonstrated their clinical utility for predicting lesion invasiveness, histologic subtype, and prognosis in lung adenocarcinoma [12].

Despite its promise (radiomics), radiomics has seen limited clinical adoption. Most models are trained on small or narrowly defined datasets and lack interpretability [13–15]. Many operate as “black boxes,” relying on complex, unintuitive features that clinicians find difficult to interpret, which does not meet the core transparency requirements for AI systems applied in clinical decision-making [16]. As a result, skepticism about transparency and clinical relevance has hindered adoption [17].

We developed a radiologic–clinical machine learning model that uses HRCT to predict pGGN invasiveness. To address the model’s “black box” limitation and improve interpretability, we applied the SHAP framework after training to quantify each feature’s contribution. SHAP is a model-agnostic, theoretically grounded method that assigns contribution values to individual features and produces visual explanations, thereby mitigating opacity and increasing clinical trust [18, 19]. Its applicability to radiomic research has been further validated by dedicated interpretation tools tailored to medical imaging scenarios [20].

We propose that a model augmented with SHAP-based post-hoc explanations can deliver accurate predictions while providing patient-specific, interpretable insights, as validated in multiple oncological domains including head and neck, brain, and prostate cancer [21–23]. By combining detailed imaging features with clinical data and applying interpretable machine-learning methods, this approach can improve the consistency and quality of clinical decisions and support the development of personalized treatment plans for patients with early-stage pulmonary adenocarcinoma.

Despite these advances, radiomics adoption remains limited by three major challenges: a lack of interpretability (the “black box” problem), limited integration with clinical data, and ongoing interobserver variability in conventional HRCT assessment.

This study addresses those gaps with three advances. First, we developed an integrated model that combines clinical variables and radiomic features to exploit their complementary information. Second, we applied the SHAP framework to quantify feature importance and to interpret individual predictions, reducing the “black box” problem of conventional machine learning models. Third, we used high-resolution targeted CT volumetric datasets to extract finer radiomic features, enhancing the model’s ability to discriminate subtle lesions.

## Methods

### Study population

The Ethics Committee of Yantai Yuhuangding Hospital approved this retrospective study (approval number: 2024 − 417).

We analyzed de-identified clinical data from a retrospective cohort of patients with pGGNs who underwent preoperative HRCT using a targeted protocol. Clinical and radiologic records were reviewed retrospectively. All detected pulmonary nodules were surgically excised and histopathologically classified within the adenocarcinoma spectrum: AAH, AIS, MIA, and IAC. Study enrollment spanned January 2019 through December 2024. The patient recruitment flowchart is provided in Supplementary Figure S9.

Eligibility criteria were: (1) presence of pGGNs on HRCT lung window settings (1500 HU width, − 600 HU level); (2) histopathological confirmation of pulmonary adenocarcinoma; and (3) surgical resection performed within seven days after CT imaging.

Exclusion criteria were as follows: (1) CT images deemed unsuitable for targeted feature extraction using AI assistance, specifically those with a voxel size greater than 1 mm, severe motion or metal artifacts, incomplete nodule coverage, or technical errors that adversely affect the accuracy of radiomic feature extraction; (2) the presence of multiple ground-glass nodules; (3) solid or part-solid nodules; (4) maximum nodule diameter exceeding 3 centimeters; (5) a history of preoperative treatment or biopsy; and (6) preoperative CT images exhibiting significant artifacts that hindered further analysis.

We applied strict exclusion criteria to improve cohort homogeneity and reduce confounders that might bias radiomic feature extraction and model performance. Specifically: (1) multiple nodules can interact and complicate lesion-to-pathology correlation; (2) solid or part-solid nodules differ pathologically and radiographically from pure GGNs; (3) nodules > 3 cm carry a much higher pretest probability of invasiveness and follow different clinical management pathways; and (4) preoperative treatment or biopsy can alter imaging characteristics. These exclusions reduced heterogeneity in the study population and thereby strengthened the internal validity of our model.

This study used a retrospective convenience cohort; the final sample size therefore reflects the number of eligible cases remaining after applying the inclusion and exclusion criteria. We assessed sample-size adequacy with two complementary, widely accepted approaches for radiomic model development. First, standard guidance for logistic regression recommends an events-to-variables ratio of at least 10:1. Our cohort comprised 148 invasive lesions and 87 noninvasive lesions, and the final model included 10 predictors, yielding an events-to-variables ratio of 14.8:1. This exceeds the recommended minimum and supports the statistical feasibility of the model. Second, prior radiomics studies of pGGN invasiveness report that cohorts of ≥ 200 patients typically produce stable, reproducible models [13, 24]. Our total cohort of 235 patients meets this benchmark. No formal a priori power calculation was performed.

### Acquisition of CT imaging

All participants underwent unenhanced (non-contrast) thoracic CT on Brilliance iCT scanners (Philips Healthcare, Best, The Netherlands). Scans were acquired supine at full inspiration, extending from the pulmonary bases to the thoracic inlet. Targeted HRCT was obtained on the same Brilliance iCT platform with 0.67 mm slice thickness, 120 kV tube voltage, and a Lung Enhanced reconstruction filter. Full scan parameters are listed in Supplementary Methods.

### Image preprocessing

We first standardized and normalized the images to reduce noise and imaging artifacts. We then discretized gray levels using a bin width of 5. Before extracting features, all samples were resampled to a uniform voxel size of 0.195 mm × 0.195 mm × 0.335 mm (all dimensions < 1 mm) to match the native resolution of our HRCT scans and preserve the fine structural details of pGGNs. Although this voxel spacing is not strictly isotropic, the slice thickness (0.335 mm) is close to the in-plane resolution (0.195 mm), and prior studies have shown that this degree of anisotropy has minimal impact on radiomic features for lung nodule analysis [9, 25].

### Segmentation of pulmonary nodules

Pulmonary nodule boundaries were delineated on HRCT datasets acquired with targeted protocols using ITK-SNAP v4.2.2 (https://www.itksnap.org/pmwiki/pmwiki.php) and optimized lung window settings (window width: 1500 HU; window center: −600 HU). An attending radiologist traced each nodule with the polygon tool in the main interface. The software then automatically generated the full tumor contour and designated it as the region of interest (ROI) on axial images.

The radiologist removed extraneous tissue using digital annotation tools, then refined the ROI by interactively editing contours and defining precise boundaries on individual axial sections. Structures adjacent to the nodule margins—including involved pleura, blood vessels, and bronchi—were excluded from the segmentation using targeted adjustments with the paintbrush tool.

### Radiomic feature extraction

Radiomic features were extracted with the PyRadiomics toolkit (version 3.1.0; https://pyradiomics.readthedocs.io/en/latest/index.html) [26]. For each lesion, 1,834 radiomic descriptors were derived from both original CT images and filtered transformed images. These features included three core categories:

1. Shape metrics (2D and 3D).
2. First-order statistics (histogram features, including CT attenuation parameters: maximum, minimum, mean, median, and standard deviation).
3. Texture measures.

To capture multi-scale structural information, we enabled five built-in image filters in PyRadiomics (consistent with the feature names used in our final model):

- Laplacian of Gaussian (LoG, log_sigma): We used a single spatial scale of σ = 2.0 mm to enhance edges and fine structures, capturing subtle variations in nodule texture associated with cellular heterogeneity.
- Wavelet transform (wavelet): Performed 1-level decomposition yielding 8 subbands (LLL, HHL, etc.), where low-frequency subbands reflect gross morphological features and high-frequency subbands capture microstructural details.
- Logarithm transform (logarithm): Compresses the dynamic range of CT values, improving the detection of low-contrast heterogeneity within pGGNs.
- Square Root transform (squareroot): Stabilizes the variance of CT intensity values, reducing the impact of extreme outliers on feature calculation.
- 3D Local Binary Pattern (lbp_3D): Quantifies local texture patterns by comparing each voxel to its 26 neighboring voxels, identifying repetitive structural features associated with invasiveness.

### Clinical characteristics and CT image evaluation

We extracted clinical parameters—age, sex, smoking history, personal history of malignancy, and family history of cancer—from patient medical records, informed by oncological studies [27, 28].

We extracted quantitative and qualitative imaging descriptors with the United Imaging AI platform (version R001.0.1.42690). Quantitative metrics included CT attenuation parameters (maximum, minimum, mean, median, and standard deviation), texture measures (kurtosis, skewness, entropy), and size/volume measures. The size/volume measures comprised total volume and total mass; the volume of components with attenuation below − 300 HU (Volume₋₃₀₀); the proportion of volume below − 300 HU (VolumePercent₋₃₀₀); the mass of components below − 300 HU (Mass₋₃₀₀); the proportion of mass below − 300 HU (MassPercent₋₃₀₀); and the maximal longitudinal (major axis) and transverse (minor axis) dimensions on axial images. We defined necrosis ratio as the proportion of nodule volume with CT attenuation below − 600 HU, representing hypoattenuating regions that may reflect microscopic necrosis in pGGNs. Although overt necrosis is uncommon in pure GGNs, subtle hypoattenuating foci have been linked to greater invasiveness in prior studies [29]. Qualitative descriptors covered attenuation pattern, spatial distribution within the pulmonary parenchyma, malignancy risk category, and architectural features such as lobulated contour, pleural retraction, bronchiolar lucency, cystic lucency, and vascular angulation with microlobulated projections. No cases showed spiculation, vascular convergence, or burr-like projections; only one case exhibited an air bronchogram, and lobulation was extremely rare. Because of their absence or extreme rarity, these features were excluded from further analysis. Two board-certified thoracic radiologists, with 8 and 10 years of diagnostic imaging experience respectively, independently performed risk stratification of pulmonary nodules following the Fleischner Society guidelines. Nodules were labeled low-risk or high-risk according to morphological criteria—size, margin characteristics, and internal structure. Interobserver agreement was excellent (Kappa = 0.87). Although this radiologic feature was entered into the univariate logistic regression, it did not emerge as an independent predictor in the multivariate analysis and therefore was excluded from the final clinical and combined models. We then performed a correlation analysis across all clinical variables. For feature clusters whose inter-feature correlation exceeded the predefined threshold (*r* > 0.90), we retained variables according to a hierarchical set of criteria: (1) established clinical relevance to pGGN invasiveness; (2) high measurement reliability (ICC > 0.85, based on our reproducibility analysis); and (3) stronger univariate association with the outcome (lower p-value). Only when multiple variables within a cluster were equivalent in clinical relevance, reliability, and association strength did we select among them at random.

### Reproducibility analysis

To assess methodological reproducibility, we randomly selected 20 participants for a verification sub-study. Two board-certified thoracic radiologists (Reader 1 and Reader 2, with 8 and 10 years of diagnostic imaging experience, respectively) independently performed semiautomated region-of-interest (ROI) **s**egmentation while blinded to patient demographics to evaluate inter-rater reliability. To measure temporal stability, the same observer repeated ROI delineation after a 30-day interval. We quantified interobserver and intraobserver agreement of radiomic feature extraction using the intraclass correlation coefficient (ICC). Radiomic features with ICC > 0.75 satisfied the predefined threshold for acceptable reproducibility and were retained for subsequent analyses.

### Clinical model development

We systematically evaluated epidemiologic factors—age, sex, tobacco exposure, prior neoplastic diagnoses, and inherited cancer susceptibility—alongside morphometric and radiomic features to develop a risk-stratification model for early cancer detection.

We constructed the clinical model using stepwise multivariate logistic regression. Candidate variables were first screened by univariate analysis, and predictors with multicollinearity (VIF > 10) were excluded. The final model retained only mean CT value and VolumePercent₋₃₀₀ as independent predictors.

### Radiomic model development

We built the radiomic and combined models with an Extremely Randomized Trees (ExtraTrees) classifier, which resists overfitting and performs well on high-dimensional radiomic data. Model development followed three steps. First, we used the Mann–Whitney U test (rank-sum test) with Bonferroni correction to identify features that differed significantly between non-invasive and invasive cohorts, retaining variables with *p* < 0.05. Second, we removed redundant features by assessing multicollinearity and excluding parameters with |r| ≥ 0.90. Third, we selected the final predictor set with L1-regularized regression (LASSO) using 10-fold cross-validation to tune the regularization parameter (λ = 0.01). After feature selection, we constructed the radiomic signature using machine learning.

We compared the diagnostic performance of the three models in the training and testing cohorts using receiver operating characteristic (ROC) analysis, reporting area under the curve (AUC), sensitivity, and specificity. We evaluated calibration by plotting calibration curves and performing the Hosmer–Lemeshow test. Finally, we applied decision curve analysis (DCA) to estimate the potential net benefit of the clinical, radiomic, and combined models in the development and testing cohorts.

### Model explanation and visualization

SHAP (SHapley Additive exPlanations) applies coalition game theory to reduce the “black-box” opacity of machine learning models by identifying and ranking the features that drive class predictions [18, 19]. The Shapley-based attribution metric measures the average incremental contribution of each feature to a model’s output by evaluating all possible feature subset permutations. It is especially useful when individual features can have outsized effects on outcomes while the overall result depends on their combined contributions [18, 19].

We used a Shapley-based kernel estimator to quantify feature importance, treating each patient characteristic as a contributing player in a cooperative game and the model output as the collective payoff. A feature’s contribution therefore directly reflects its clinical relevance for the model’s prediction.

## Results

### Patient demographic characteristics

We retrospectively enrolled 235 pGGNs from 235 patients according to the prespecified inclusion and exclusion criteria. The lesions were randomly assigned to training and testing cohorts at a 7:3 ratio. The training cohort included 64 pre-invasive and 100 invasive lesions, while the testing cohort comprised 23 pre-invasive and 48 invasive lesions. Baseline demographics for both cohorts are summarized in Table 1.

**Table 1:** Demographic, clinical, and imaging characteristics of the training and testing cohorts (N(%))

	Training cohort				Testing cohort			
	ALL *N =* 164	Non-invasive *N =* 64	Invasive *N =* 100	*P* value	ALL *N* = 71	Non-invasive *N =* 23	Invasive *N =* 48	*P* value
Age (years, mean ± SD)	52.05 ± 13.40	52.98 ± 12.31	51.45 ± 14.08	0.594	51.56 ± 11.36	48.17 ± 8.67	53.19 ± 12.19	0.082
Maximum CT value (HU, mean ± SD)	-373.66 ± 172.41	-421.33 ± 177.21	-343.15 ± 162.93	0.003	-368.54 ± 165.94	-468.43 ± 136.60	-320.67 ± 158.28	< 0.001
Minimum CT value	-729.89 ± 115.42	-744.47 ± 116.75	-720.56 ± 114.18	0.197	-711.18 ± 107.94	-737.39 ± 93.09	-698.62 ± 113.13	0.158
Mean CT value	-585.71 ± 111.31	-620.93 ± 100.79	-563.17 ± 112.34	0.001	-564.82 ± 111.80	-615.30 ± 95.37	-540.63 ± 111.87	0.007
CT value standard deviation	68.71 ± 32.70	63.79 ± 37.21	71.85 ± 29.23	0.030	67.47 ± 33.61	54.73 ± 26.36	73.58 ± 35.21	0.011
Kurtosis	3.80 ± 2.56	3.81 ± 2.27	3.79 ± 2.74	0.968	3.53 ± 1.96	3.46 ± 2.01	3.56 ± 1.95	0.749
Skewness	0.47 ± 0.81	0.48 ± 0.83	0.47 ± 0.80	0.782	0.43 ± 0.66	0.38 ± 0.65	0.46 ± 0.67	0.653
Entropy	6.17 ± 1.44	5.79 ± 1.55	6.42 ± 1.32	0.014	6.12 ± 1.37	5.66 ± 1.11	6.33 ± 1.43	0.052
Overall volume (mm³, mean ± SD)	433.28 ± 622.77	286.60 ± 316.25	527.16 ± 743.05	0.002	360.85 ± 522.49	167.76 ± 126.36	453.38 ± 610.02	0.003
Volumepercent₋₃₀₀ (%, mean ± SD)	96.75 ± 9.75	98.48 ± 5.38	95.64 ± 11.61	0.035	96.31 ± 11.28	98.41 ± 6.93	95.31 ± 12.80	0.031
Long diameter (mm, mean ± SD)	8.70 ± 3.80	7.67 ± 2.71	9.36 ± 4.24	0.005	8.14 ± 3.45	6.43 ± 1.24	8.96 ± 3.86	< 0.001
Sex				0.828				0.866
Female	115(70.12)	46(71.88)	69(69.00)		50(70.42)	17(73.91)	33(68.75)	
Male	49(29.88)	18(28.12)	31(31.00)		21(29.58)	6(26.09)	15(31.25)	
Smoking history				0.237				0.847
No	135(82.32)	56(87.50)	79(79.00)		55(77.46)	17(73.91)	38(79.17)	
Yes	29(17.68)	8(12.50)	21(21.00)		16(22.54)	6(26.09)	10(20.83)	
Personal tumor history				0.674				0.821
No	159(96.95)	63(98.44)	96(96.00)		69(97.18)	23(100.00)	46(95.83)	
Yes	5(3.05)	1(1.56)	4(4.00)		2(2.82)	0(0.00)	2(4.17)	
Family cancer history				0.042				0.058
No	147(89.63)	53(82.81)	94(94.00)		64(90.14)	18(78.26)	46(95.83)	
Yes	17(10.37)	11(17.19)	6(6.00)		7(9.86)	5(21.74)	2(4.17)	
Location				0.331				0.375
LU	47(28.66)	21(32.81)	26(26.00)		15(21.13)	7(30.43)	8(16.67)	
LL	29(17.68)	8(12.50)	21(21.00)		16(22.54)	4(17.39)	12(25.00)	
RU	53(32.32)	21(32.81)	32(32.00)		27(38.03)	6(26.09)	21(43.75)	
RM	10(6.10)	6(9.38)	4(4.00)		4(5.63)	2(8.70)	2(4.17)	
RL	25(15.24)	8(12.50)	17(17.00)		9(12.68)	4(17.39)	5(10.42)	
Pleural indentation				0.024				0.093
No	143(87.20)	61(95.31)	82(82.00)		63(88.73)	23(100.00)	40(83.33)	
Yes	21(12.80)	3(4.69)	18(18.00)		8(11.27)	0(0.00)	8(16.67)	
Vacuole sign				0.553				1.000
No	150(91.46)	57(89.06)	93(93.00)		65(91.55)	21(91.30)	44(91.67)	
Yes	14(8.54)	7(10.94)	7(7.00)		6(8.45)	2(8.70)	4(8.33)	
Risk stratification				0.033				0.979
Low risk	45(27.44)	24(37.50)	21(21.00)		23(32.39)	8(34.78)	15(31.25)	
High risk	119(72.56)	40(62.50)	79(79.00)		48(67.61)	15(65.22)	33(68.75)	

LU: Left upper lobe; LL: Left lower lobe; RU: Right upper lobe; RM: Right middle lobe; RL: Right lower lobe Risk stratification was performed by two board-certified thoracic radiologists according to Fleischner Society guidelines. This feature was not included in the final prediction models

### Feature selection

Variables with statistical significance in univariate logistic regression were included in multivariable logistic regression to identify independent predictors. We identified Mean CT value and VolumePercent₋₃₀₀ as independent predictive variables and further used them to build the clinical predictive model. Detailed results are presented in Table 2, with the forest plot shown in Supplementary Figure S1.

Of the 1,834 extracted radiomic features, 1,090 differed significantly between groups (Mann–Whitney U test, *p* < 0.05). After removing collinear features (*r* > 0.9), 183 descriptors remained for LASSO selection. We applied the Least Absolute Shrinkage and Selection Operator (LASSO) with 10-fold cross-validation and identified the optimal regularization parameter λ = 0.0596. LASSO yielded eight nonzero coefficients that comprised the final radiomic signature: log_sigma_2_0mm_3D_firstorder_Range; squareroot_firstorder_InterquartileRange; lbp_3D_k_firstorder_Skewness; wavelet_HHL_firstorder_Mean; lbp_3D_k_firstorder_Minimum; lbp_3D_m2_firstorder_Skewness; logarithm_glszm_GrayLevelNonUniformityNormalized; and wavelet_LLL_Coarseness. The feature distributions and the LASSO selection path are shown in Supplementary Figures S7 and S8, respectively, and histograms of sample predictions appear in Supplementary Figure S3.

**Table 2:** Univariate and multivariate logistic analysis

Variable	Univariate logistic analysis			Multivariate logistic analysis		
	OR	95%CI	*P* value	OR	95%CI	*P* value
Family cancer history	0.545	0.237-1.257	0.232			
Minimum CT value	0.999	0.999-1.000	0.010	0.995	0.986-1.004	0.381
Mean CT value	0.999	0.999-1.000	0.031	1.012	1.003-1.022	0.029
Maximum CT value	1.000	0.999-1.000	0.164			
Vacuole	1.000	0.415-2.408	1.000			
Overall Volume	1.001	1.001-1.002	0.001	1.001	0.999-1.003	0.417
Volumepercent₋₃₀₀	1.004	1.002-1.007	0.009	1.035	1.006-1.066	0.048
CT value standard deviation	1.007	1.003-1.010	0.002	0.983	0.966-1.001	0.128
Age	1.008	1.003-1.012	0.011	0.993	0.970-1.016	0.608
Long diameter	1.066	1.035-1.097	<0.000	1.057	0.801-1.395	0.742
Kurtosis	1.087	1.022-1.156	0.024	0.975	0.848-1.120	0.760
Entropy	1.088	1.043-1.135	0.001	1.217	0.814-1.820	0.422
Location	1.157	1.057-1.267	0.008	1.013	0.803-1.276	0.929
Risk stratification	1.235	1.114-1.369	0.001	1.194	0.802-1.779	0.463
Skewness	1.255	0.946-1.664	0.185			
Sex	1.381	1.138-1.675	0.006	0.923	0.456-1.868	0.851
Smoking history	2.625	1.326-5.197	0.020	1.567	0.668-3.677	0.386
PersonalTumorHistory	4.000	0.636-25.154	0.215			
Pleural indentation	6.000	2.151-16.743	0.004	2.249	0.711-7.114	0.247

### Performance of clinical, radiomic, and combined models

**Fig. 1:**
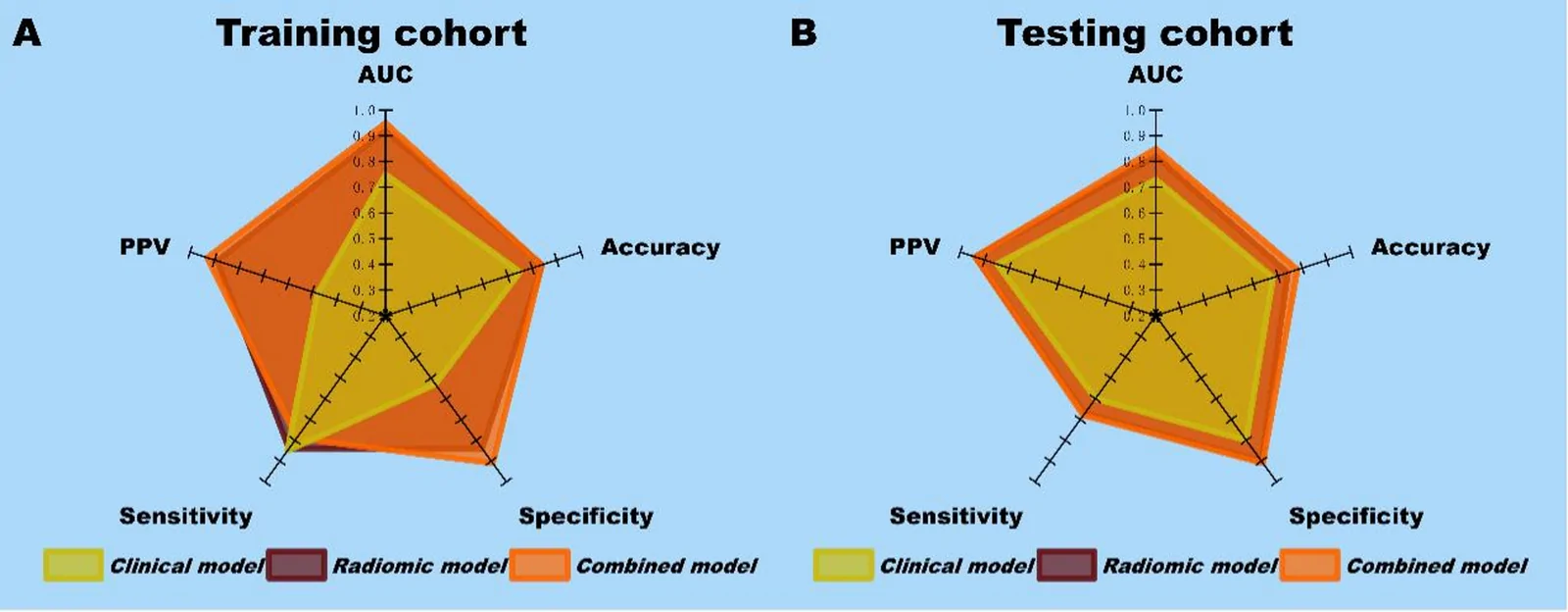
Receiver operating characteristic (ROC) curves of the clinical, radiomic, and combined models in the training and testing cohorts. (**A**) training cohort; (**B**) testing cohort

Figure 1 presents ROC curves and AUC values for the three models in both cohorts. The combined model performed comparably to, or slightly better than, the radiomic model, and both exceeded the clinical model. In the training set, ROC-AUCs were 0.923 (combined), 0.917 (radiomic), and 0.799 (clinical); in the testing cohort, they were 0.832, 0.827, and 0.733, respectively. Complete performance metrics for all models appear in Table 3.

**Table 3:** Diagnostic performance of three prediction of GGN invasiveness

Cohort	Model	Accuracy	AUC	95% CI	Sensitivity	Specificity	PPV	NPV
Training cohort	Clinical model	0.750	0.799	0.7324 - 0.8663	0.890	0.531	0.748	0.756
	Radiomic model	0.841	0.917	0.8761 - 0.9582	0.840	0.844	0.894	0.771
	Combined model	0.835	0.923	0.8840 - 0.9620	0.790	0.906	0.929	0.734
Testing cohort	Clinical model	0.676	0.733	0.6126 - 0.8539	0.604	0.826	0.879	0.500
	Radiomic model	0.746	0.827	0.7246 - 0.9294	0.667	0.913	0.941	0.568
	Combined model	0.761	0.832	0.7360 - 0.9288	0.687	0.913	0.943	0.583

Notably, the combined model’s sensitivity fell from 0.79 in the training cohort to 0.69 in the testing cohort, while specificity remained essentially unchanged (0.906 vs. 0.913). This pattern indicates the model more reliably identifies non-invasive pGGNs than invasive ones. The decline in sensitivity may reflect the testing cohort’s smaller sample size and the intrinsic heterogeneity of invasive lesions. Precision–recall curves (Supplementary Figure S6) were generated to further assess this performance imbalance. The combined model achieved AUC-PR values of 0.941 in the training cohort and 0.857 in the testing cohort.

DeLong’s test was used to compare AUC values between models. In the training cohort, the combined and radiomic models each significantly outperformed the clinical model (both *p* < 0.001), while the difference between the combined and radiomic models was not significant (*p* = 0.359). In the testing cohort, no pairwise differences reached statistical significance (clinical vs. radiomics: *p* = 0.111; clinical vs. combined: *p* = 0.121; radiomics vs. combined: *p* = 0.672). Detailed pairwise comparisons are shown in Supplementary Figure S2.

**Fig. 2:**
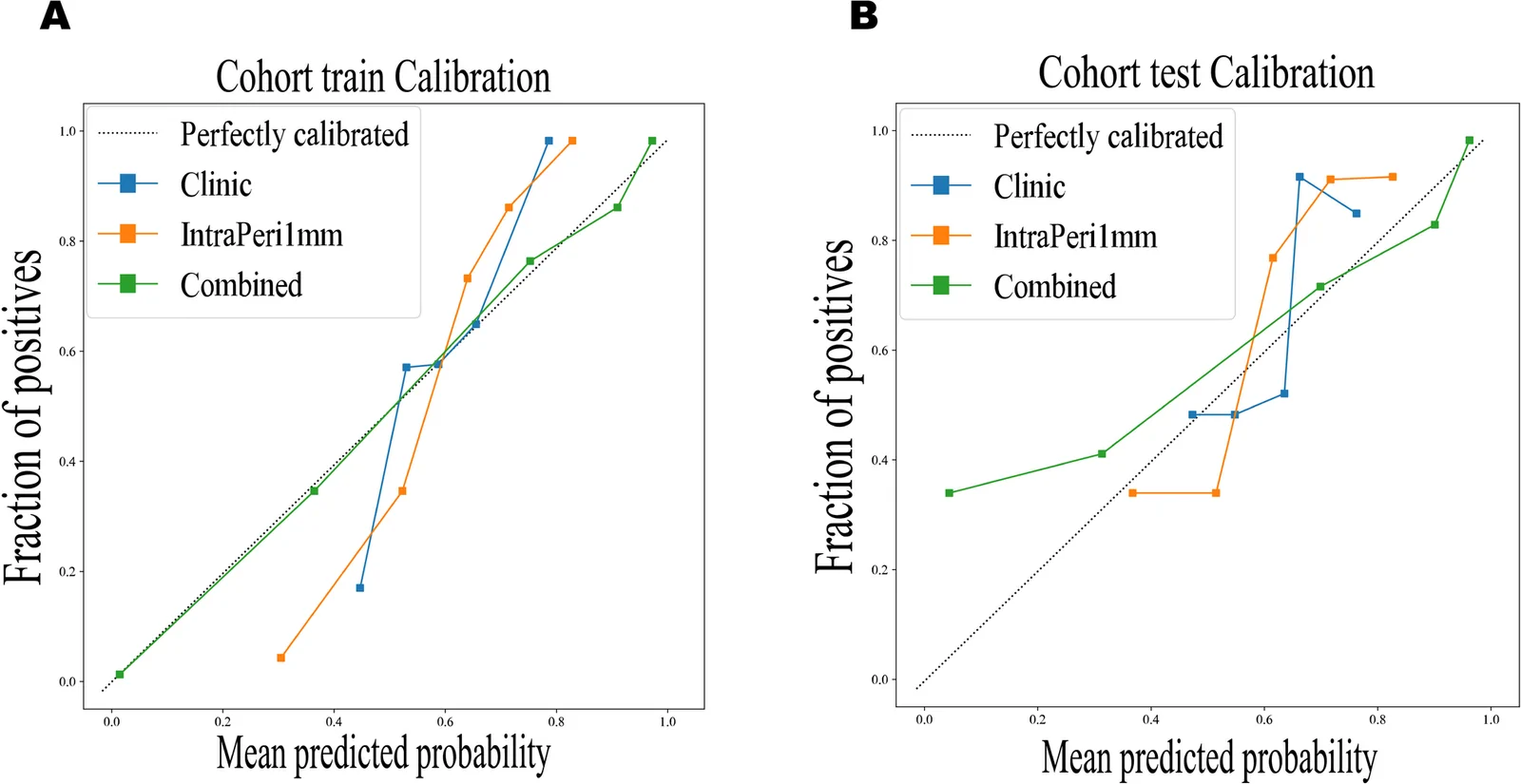
Calibration curves of the three models in the training and testing cohorts. (**A**) training cohort; (**B**) testing cohort

Figure 2 presents calibration curves showing close agreement between predicted and observed outcomes in both the training and testing datasets. In each dataset, the observed and ideal prediction curves closely overlap, indicating good calibration for all three models—clinical, radiomic, and combined.

We performed decision curve analysis (DCA) to assess the clinical utility of the three models. In the testing cohort, DeLong’s test found no statistically significant differences among the models. Nevertheless, across most threshold probabilities (0.1–0.8) the combined and radiomic models produced higher net benefits than the clinical model. Focusing on the clinically relevant range for surgical decision-making (0.3–0.6), the combined model achieved a net benefit of 0.35 at threshold = 0.4, compared with 0.32 for the radiomic model and 0.21 for the clinical model. These results suggest the combined model more effectively identifies true-positive invasive lesions while reducing unnecessary interventions for noninvasive lesions. Full DCA curves are shown in Supplementary Figure S10.

Supplementary clinical utility curves (Supplementary Figure S5) further demonstrate that the combined model reduces false-positive interventions by 52% compared to the clinical model at a decision threshold of 0.4.

**Fig. 3:**
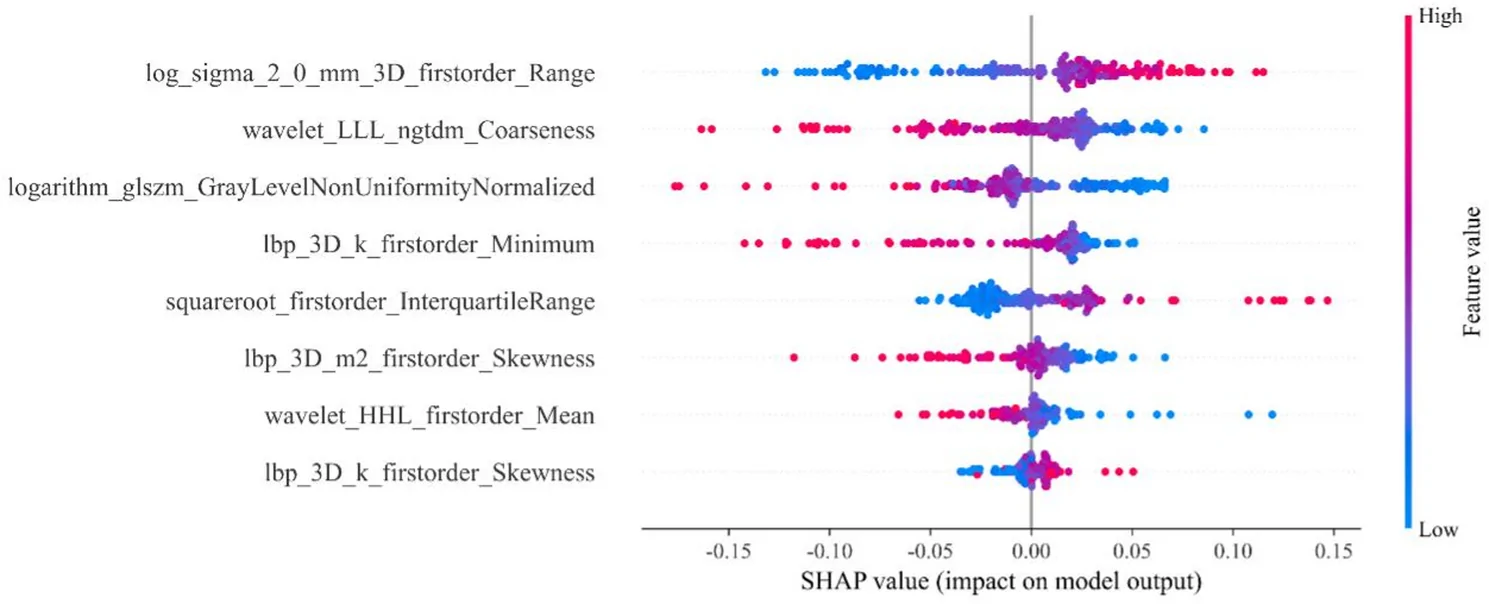
presents a SHAP beeswarm plot for the integrated model, highlighting feature importance and the contributions of multimodal inputs to predictive performance

We used SHAP (SHapley Additive exPlanations) to quantify feature contributions in the ExtraTrees model. SHAP beeswarm plots compactly display each feature’s range and dispersion of contributions to individual predictions and link feature values to their directional influence. Features are ranked by their overall importance. In the plot, each point represents the SHAP value of a given feature for one subject; vertically aligned points indicate clusters of similar SHAP values, and the color gradient (blue to red) encodes the underlying feature magnitude. In Fig. 3, log_sigma_2_0mm_3D_firstorder_Range emerged as the single most important feature for discriminating preinvasive from invasive adenocarcinoma. Its density of SHAP values across the cohort shows substantial variability, and the color trend indicates that higher values of this feature drive larger positive model outputs.

SHAP force plots (Supplementary Figure S11) visually explain model predictions by showing how individual radiomic features shift each patient’s classification from a baseline value—the mean model output across the dataset. Features appear as directional arrows: red arrows indicate positive contributions and blue arrows indicate negative contributions to the final prediction. Arrow length represents the magnitude of each feature’s effect on the SHAP value.

Supplementary Figures S11A and S11B present two representative cases illustrating the model’s interpretability in distinguishing non-invasive from invasive adenocarcinomas. In Supplementary Figure S11A, the SHAP value (0.31) lies below the baseline prediction, indicating a higher likelihood of non-invasive adenocarcinoma. The main contributing features—3D_firstorder_Range (− 1.0951), square root_firstorder_InterquartileRange (− 1.0213), and logarithm_glszm_GrayLevelNonUniformityNormalized (1.8458)—all decreased the probability of invasive disease in the final classification. By contrast, Supplementary Figure S11B shows a SHAP value of 0.75, above the baseline and implying a greater probability of invasive adenocarcinoma. Its principal contributors—3D_firstorder_Range (1.3867), square root_firstorder_InterquartileRange (0.3964), and k_firstorder_Minimum (0.6282)—all increased the predicted likelihood of invasiveness. SHAP waterfall plots are provided in Supplementary Figure S4.

These examples illustrate how SHAP analysis yields transparent, feature-level explanations for individual classification decisions.

## Discussion

This retrospective analysis demonstrates that integrating radiomic features with clinical parameters improves preoperative prediction of invasiveness in pGGNs associated with lung adenocarcinoma. The multimodal model outperformed models based solely on clinical or radiomic data, indicating that combining data types enhances diagnostic accuracy and supports clinical decision-making. These findings align with prior reports on the advantages of multimodal approaches in oncologic imaging [13, 24].

### Comparison with recent literature

Our results align with recent work highlighting the advantage of integrated clinical–radiomic models [13, 24]. The combined model attained a higher test AUC (0.832) than earlier radiomic reports (0.78–0.81) [13, 24], which likely reflects our use of high-resolution 0.67 mm CT slices and SHAP-guided feature selection. Importantly, and unlike most prior studies, we provide comprehensive SHAP-based explanations for individual predictions, addressing a key barrier to clinical adoption.

A major strength of this study is the use of targeted HRCT with 0.67 mm slice thickness and near-isotropic voxel sampling, which permits extraction of finer radiomic features than conventional low-resolution CT (typically 1–5 mm slice thickness). Prior low-resolution CT studies relied largely on coarse morphological measures such as nodule size and mean CT value and reported validation AUCs of 0.78–0.81 [13, 24, 30]. By contrast, our top feature, log_sigma_2_0mm_3D_firstorder_Range, quantifies intralesional heterogeneity at the ~ 2 mm scale—information that would be lost with thicker slices. Incorporating these high-frequency texture features from HRCT therefore contributed to the higher validation AUC of 0.832 in our cohort.

### Key findings and major contributions

This study produced three main findings. First, a combined model integrating clinical and radiomic features outperformed single-modality models, demonstrating the complementary value of multimodal data. SHAP analysis quantified each feature’s direction and magnitude of contribution to individual predictions but did not by itself provide clinical interpretability. To bridge this gap, we prioritized features with established clinical relevance (e.g., mean CT value) and related the top-performing radiomic features to known pathological hallmarks of pGGN invasiveness. For instance, the leading feature, log_sigma_2_0mm_3D_firstorder_Range, captures intralesional attenuation heterogeneity, which is associated with higher cellular density and greater invasive potential in adenocarcinoma. Second, log_sigma_2_0mm_3D_firstorder_Range was the strongest predictor, highlighting the importance of intralesional heterogeneity for assessing invasiveness. Third, applying the SHAP framework produced transparent, case-level explanations of model outputs, helping to bridge the gap between AI predictions and clinical practice.

The main contributions are: (1) an interpretable model that predicts pGGN invasiveness with an AUC of 0.832 in the testing cohort; (2) validation that high-resolution targeted CT can extract precise radiomic features from subtle lesions; and (3) a potential clinical decision-support tool that both predicts invasiveness and provides an explanation for each prediction, which requires further prospective validation.

### Strengths of the study

This study’s strengths are threefold. First, it uses rigorous methods—LASSO feature selection, 10-fold cross-validation, and comprehensive performance evaluation—to reduce overfitting and ensure robust, generalizable results. Second, it analyzes high-quality targeted 0.67 mm HRCT scans, allowing extraction of fine-grained radiomic features that improve sensitivity to subtle imaging signals. Third, model interpretability is enhanced through SHAP analysis, which clarifies feature contributions and supports clinical trust and adoption.

### Limitations and future directions

This study has several limitations. First, its single-center, retrospective design may limit external validity; multi-center prospective studies with more diverse populations are needed to validate the model. The testing cohort was relatively small (*n* = 71), which may have reduced statistical power to detect differences between the radiomic and combined models. A further major limitation is the highly selected cohort: we included only patients with a single pure ground-glass nodule (pGGN) and excluded those with multiple nodules, solid or part-solid nodules, or nodules larger than 3 cm. This selection bias substantially restricts the model’s generalizability to routine clinical practice, where patients frequently present with multiple or larger pulmonary lesions. Second, uncommon qualitative features such as vascular convergence and pleural retraction were absent or extremely rare in our cohort and thus not included, which may have reduced the model’s comprehensiveness. Including peritumoral characteristics—recent radiomic studies have shown their predictive value—could improve model accuracy [31, 32]. Third, we did not compare our approach with deep learning methods (for example, convolutional neural networks), which have demonstrated strong performance in medical image analysis. Future work will focus on three priorities: (1) multicenter prospective validation; (2) integration of peritumoral features, liquid biomarkers, and deep learning techniques; and (3) development of a user-friendly clinical decision-support system.

In conclusion, integrating radiomic and clinical features yields a robust, interpretable framework for assessing pGGN invasiveness in lung adenocarcinoma. Although the model performed well in this retrospective cohort, prospective validation in larger, multicenter cohorts is necessary before routine clinical adoption. Using SHAP improves transparency of model predictions and strengthens confidence in their clinical utility. Continued validation and refinement in prospective, large-scale cohorts are therefore required to advance these models toward routine use in personalized thoracic oncology.

## Electronic Supplementary Material

Below is the link to the electronic supplementary material.

Supplementary Material 1.

## Data Availability

The research datasets and materials used in this study are available from the corresponding investigator on justified request, pending approval by the institutional review board and any relevant data-sharing authorities. Because of ethical and legal restrictions on identifiable patient information, raw data cannot be made publicly available. To promote transparency and reproducibility, the main manuscript and supplementary materials provide a detailed account of the experimental design, analytical methods, and principal findings.
