# Supplementary material for: Radiomic model with SHAP-based interpretability for predicting invasiveness of pure ground-glass nodules: a retrospective study based on high-resolution computed tomography (HRCT) volumetric datasets
Source: J Cardiothorac Surg. 2026 Jul 18;21:778. doi: 10.1186/s13019-026-04560-5 (PMC13663225; doi:10.1186/s13019-026-04560-5)
Supplement: Supplementary file 1 [file 13019_2026_4560_MOESM1_ESM.pdf]

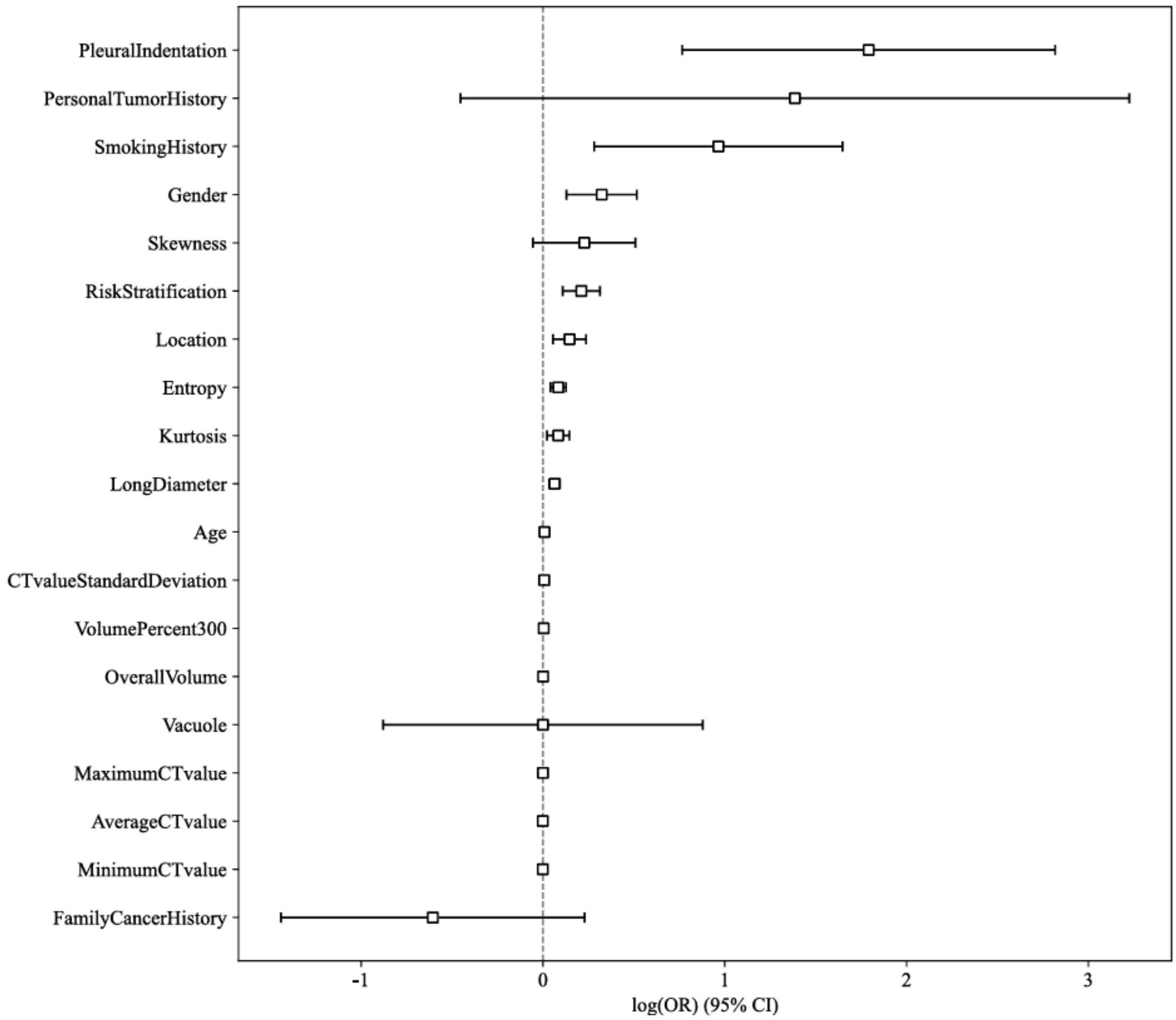

Supplementary Figure S1A. Forest plot of univariate logistic regression analysis, showing the association between clinical/radiological features and pGGN invasiveness.

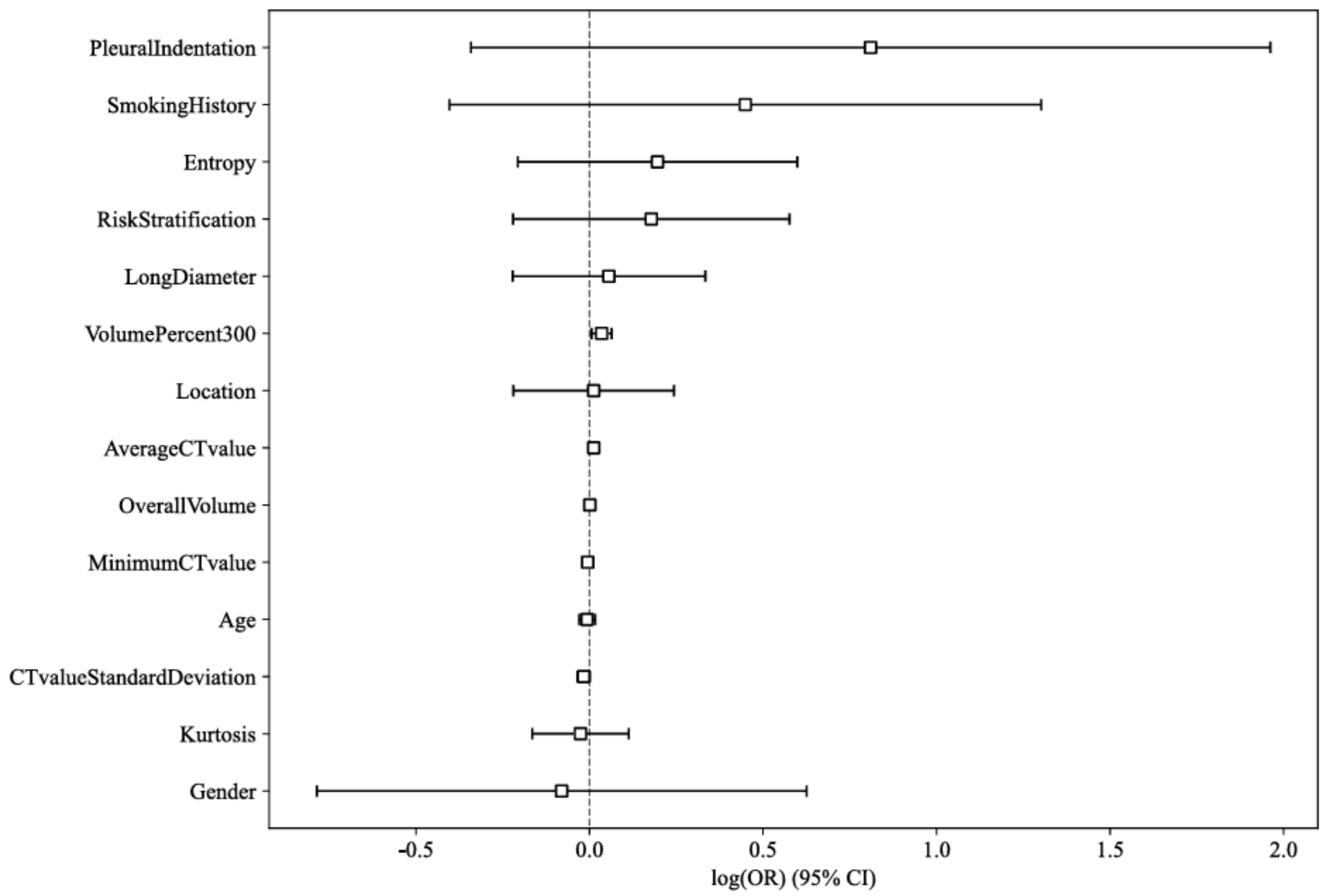

Supplementary Figure S1B. Forest plot of multivariate logistic regression analysis, showing odds ratios (OR) and 95% confidence intervals (CI) of independent predictors.

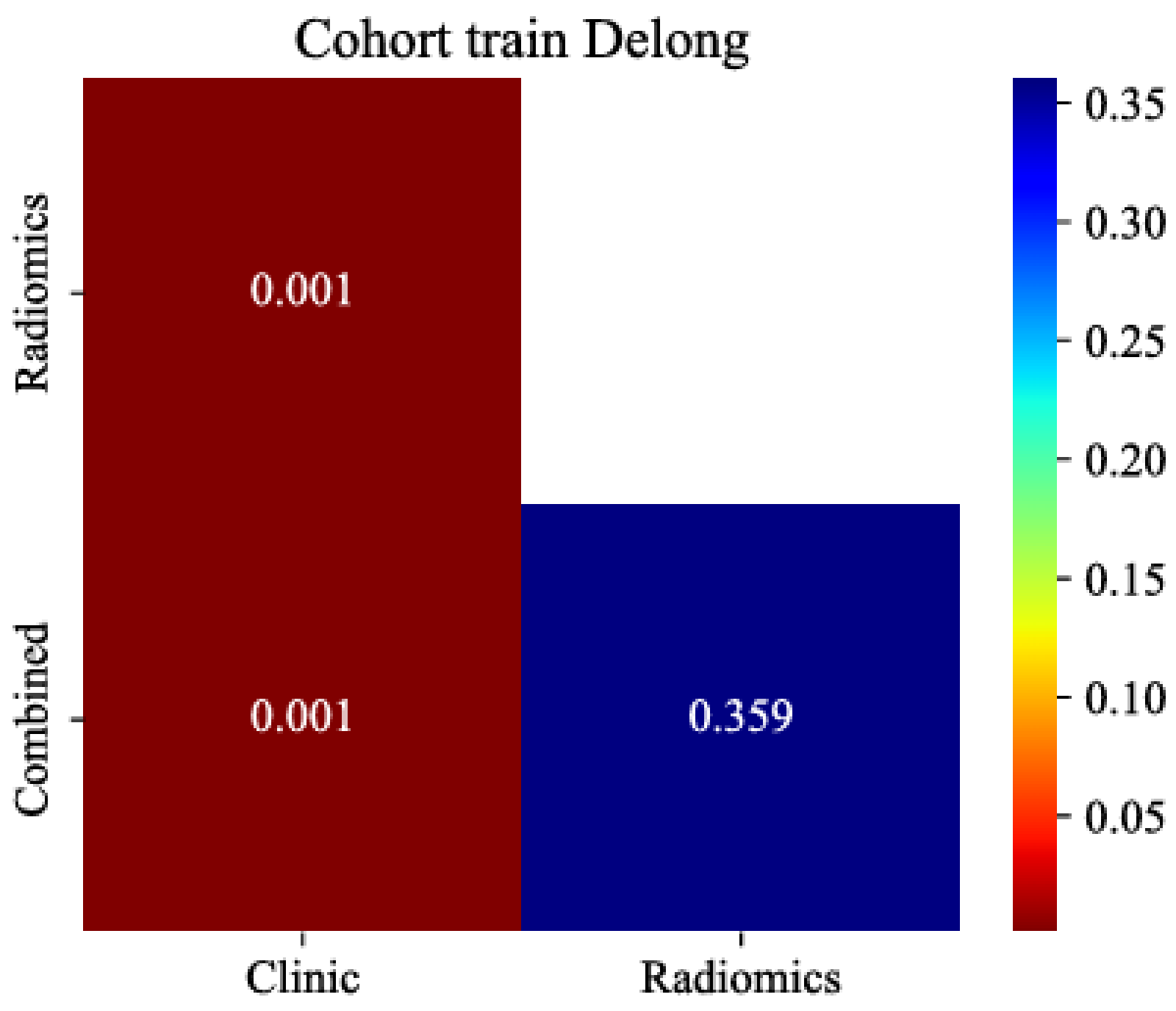

Supplementary Figure S2A. P-value matrix of DeLong's test for pairwise comparison of AUC values among the three models in the training cohort.

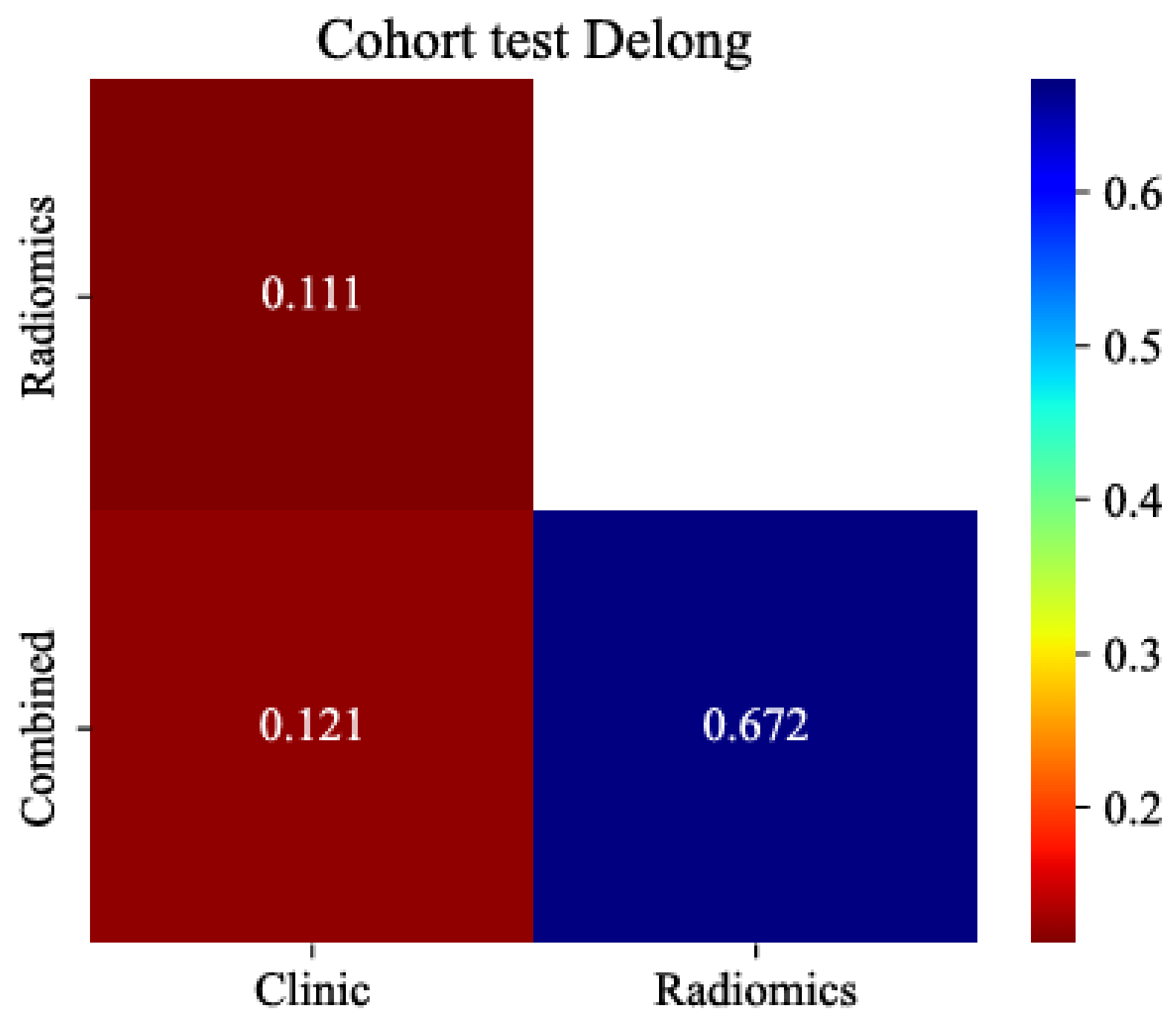

Supplementary Figure S2B. P-value matrix of DeLong's test for pairwise comparison of AUC values among the three models in the testing cohort.

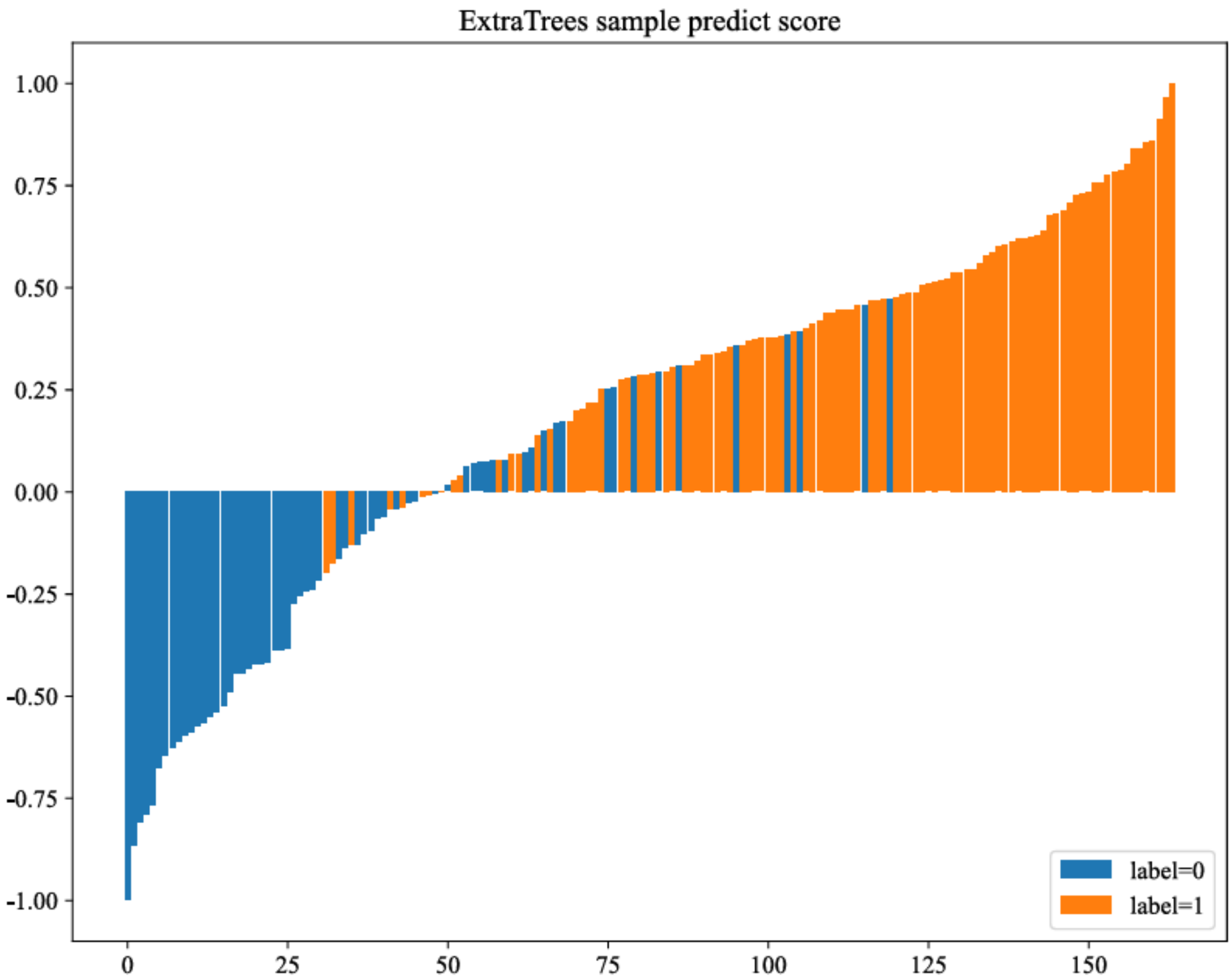

Supplementary Figure S3A. Distribution of sample prediction scores from the ExtraTrees radiomic model in the training cohort.

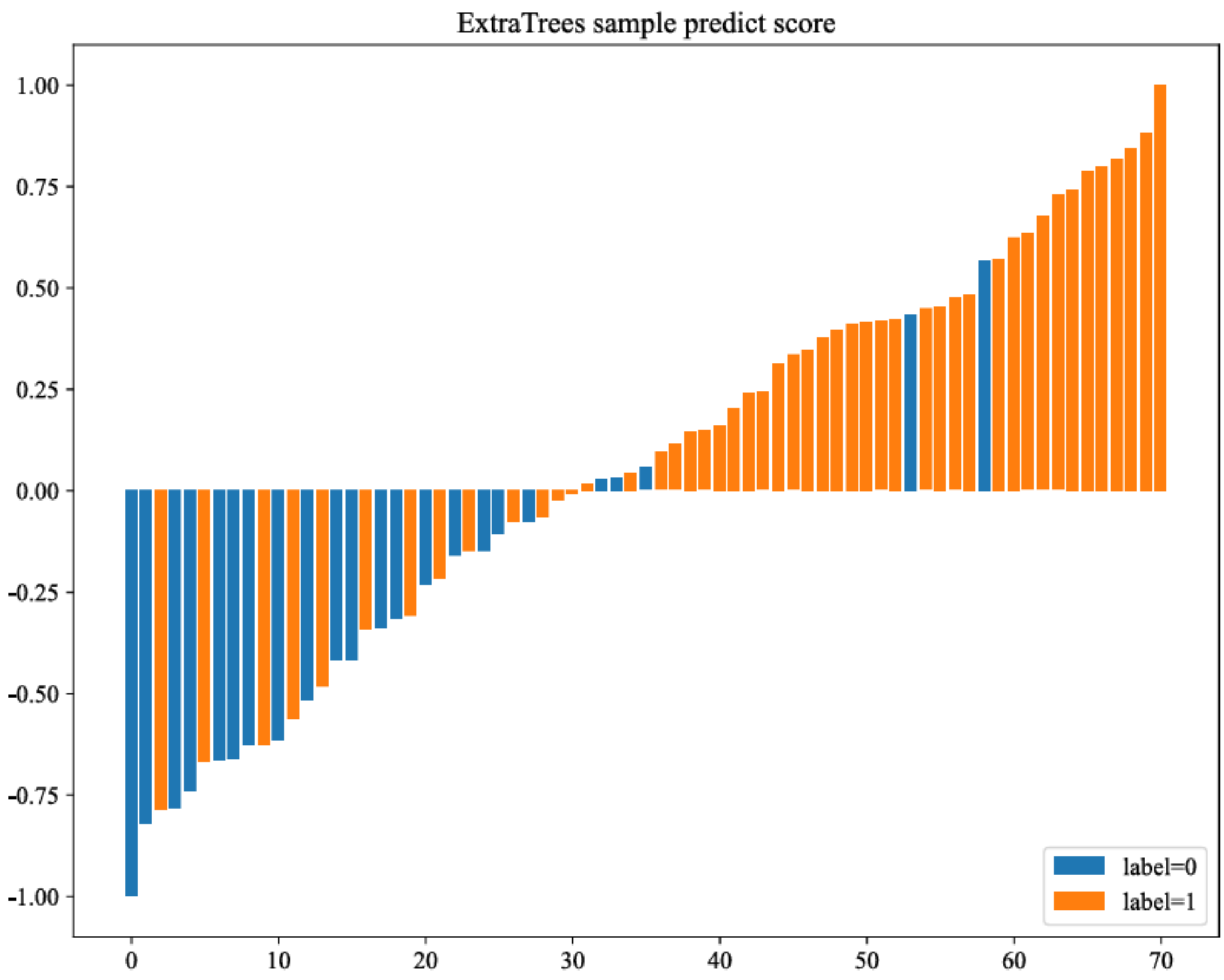

Supplementary Figure S3B. Distribution of sample prediction scores from the ExtraTrees radiomic model in the testing cohort.

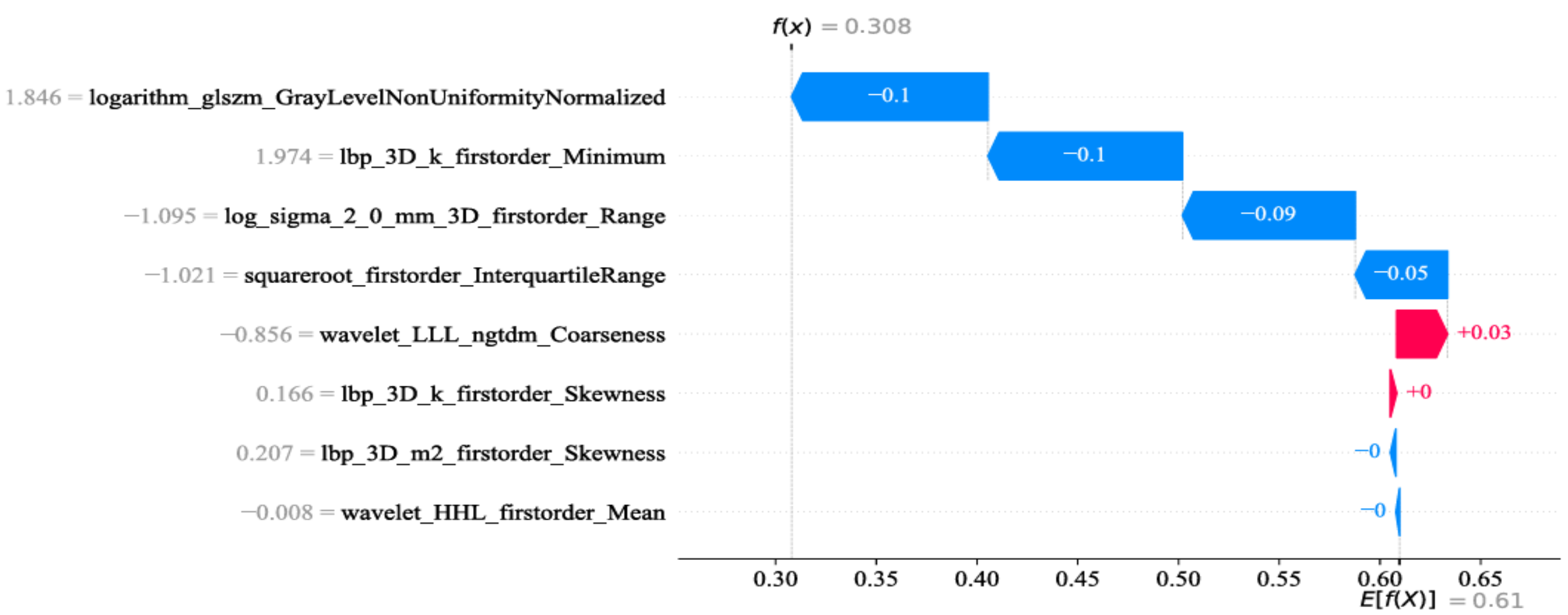

Supplementary Figure S4A. SHAP waterfall plot for a representative non-invasive pGGN case, showing the contribution of each feature to the prediction result.

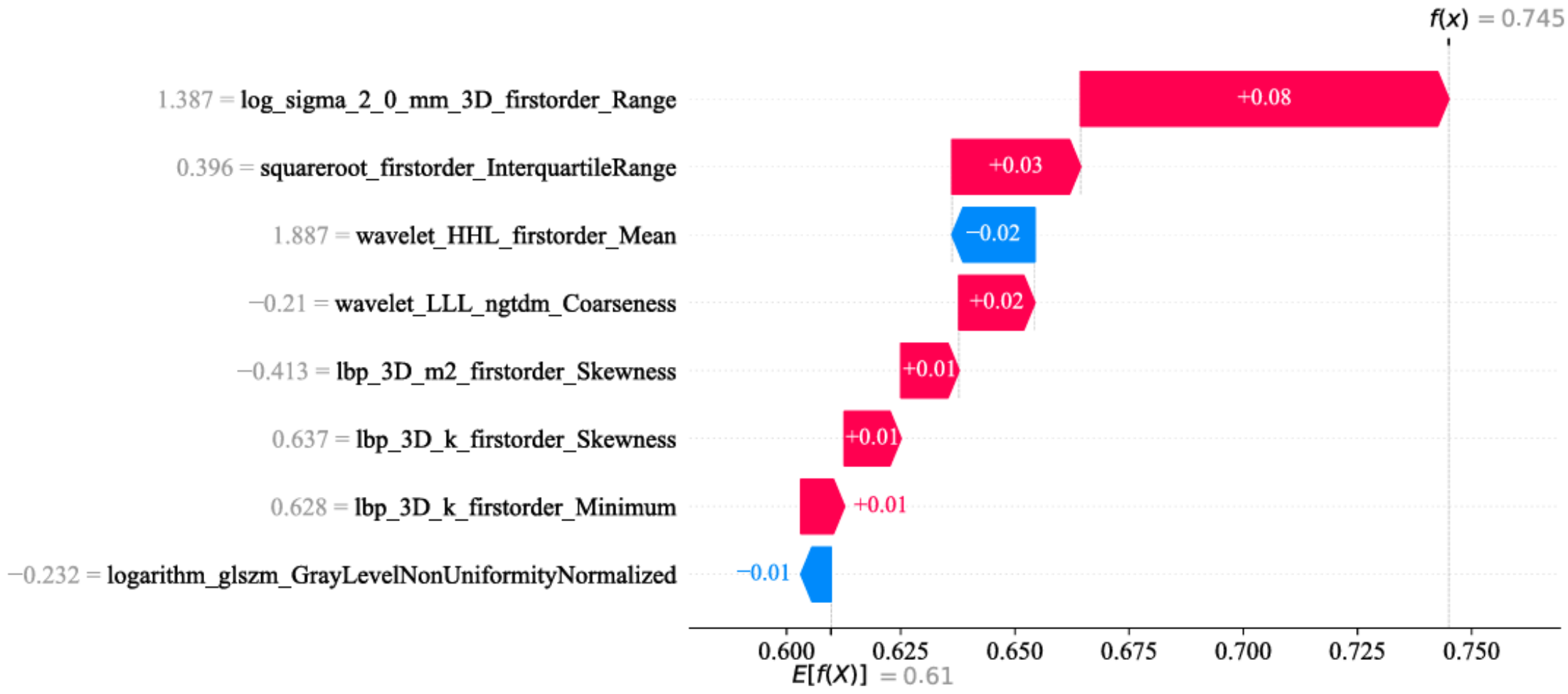

Supplementary Figure S4B. SHAP waterfall plot for a representative invasive pGGN case, showing the contribution of each feature to the prediction result.

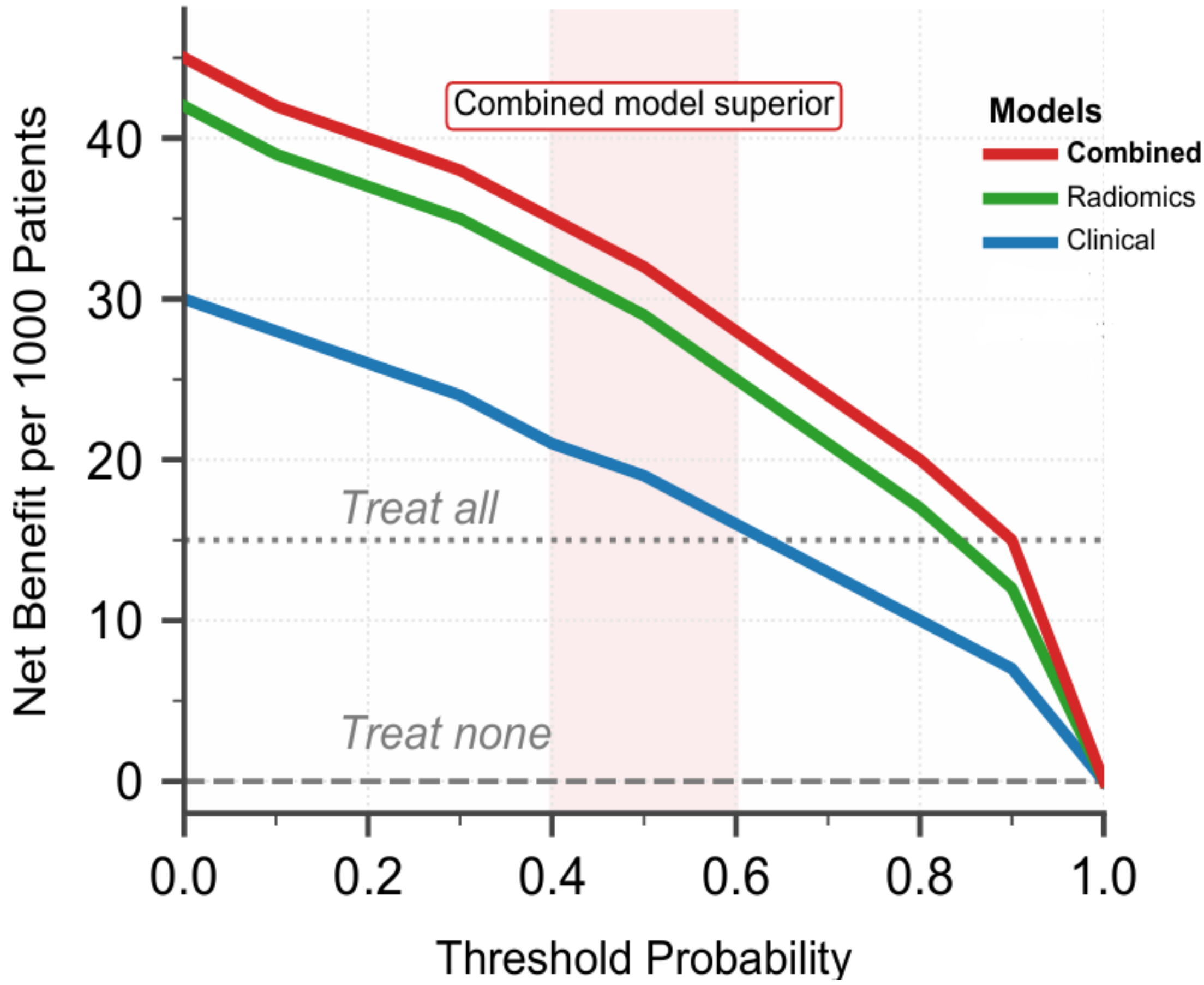

Supplementary Figure S5. Clinical utility comparison curves, showing the net benefit of the three models across different decision thresholds.

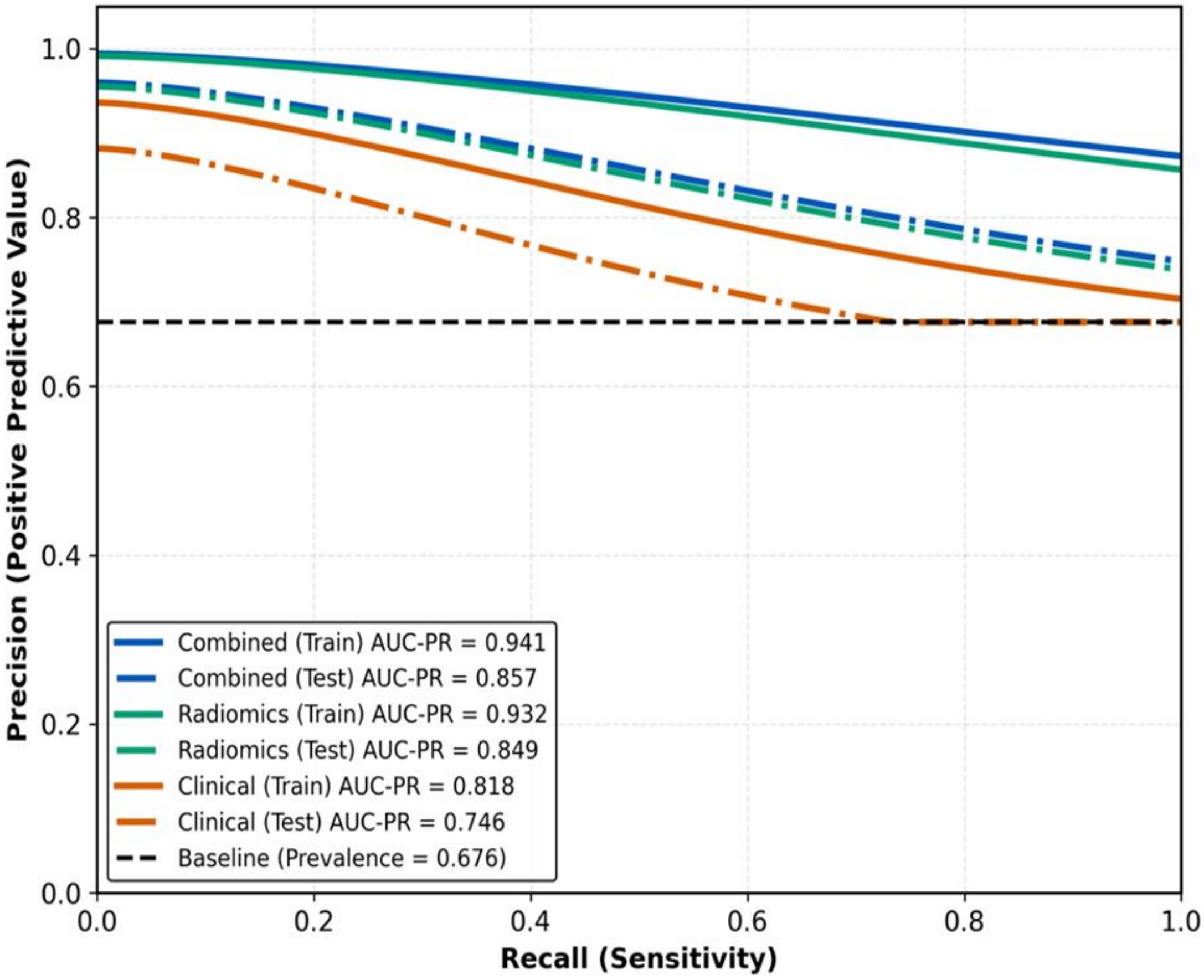

Supplementary Figure S6. Precision-recall (PR) curves of the three models in the training and testing cohorts, with corresponding AUC-PR values.

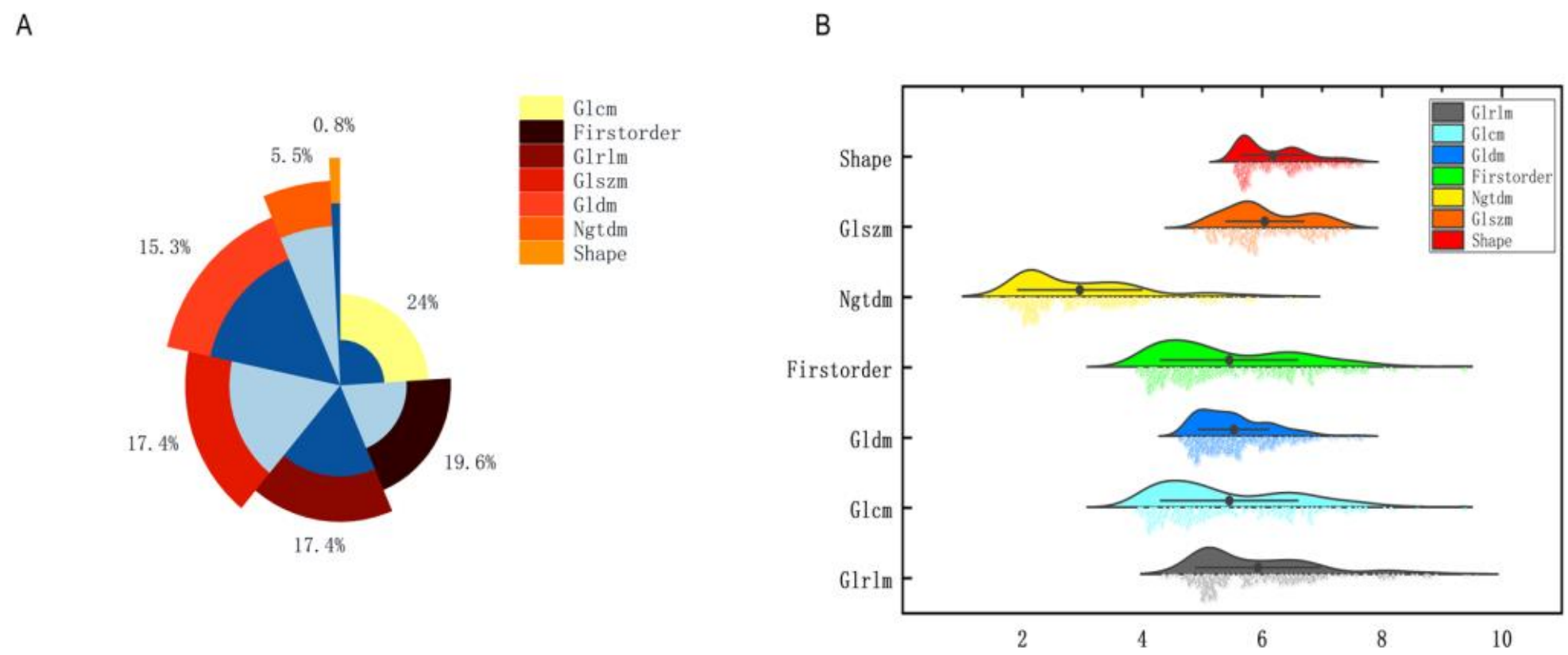

Supplementary Figure S7. Distribution of extracted radiomic features: (A) Pie chart showing the proportion of different feature categories; (B) Bar chart showing the number of features in each category.

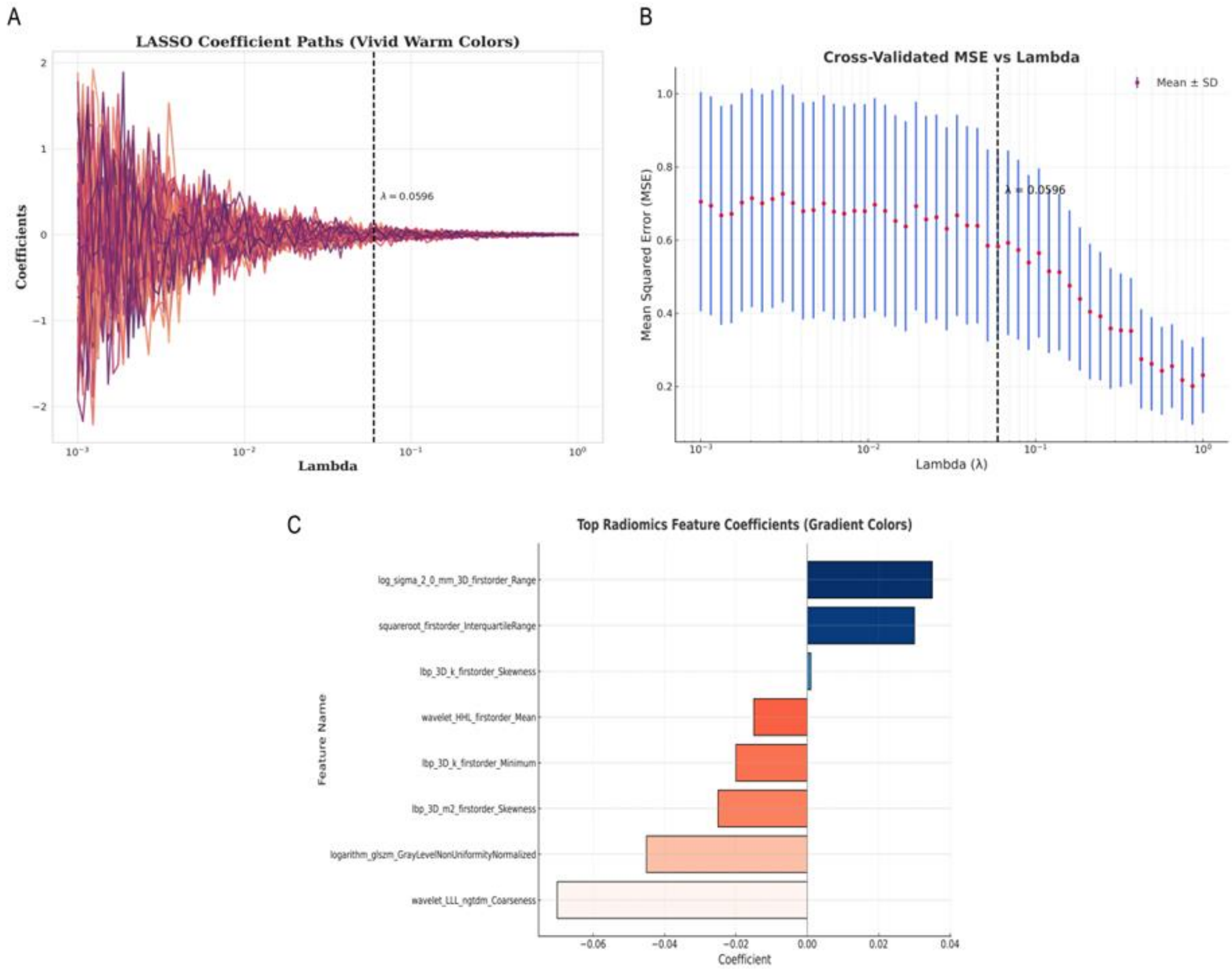

Supplementary Figure S8. LASSO regression feature selection process: (A) Feature coefficient paths; (B) Cross-validated mean squared error (MSE) versus regularization parameter λ; (C) Coefficient values of the 8 final selected radiomic features.

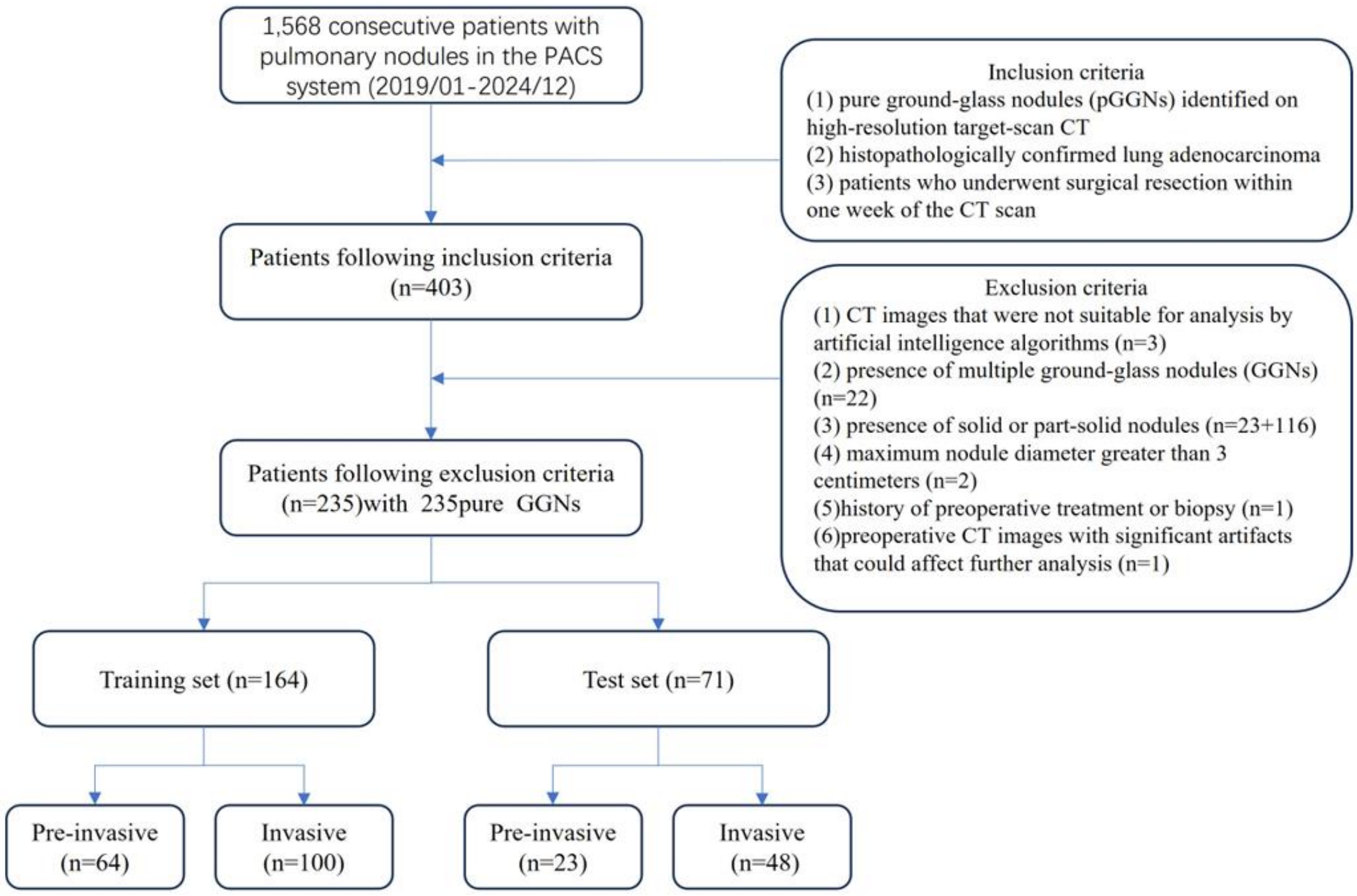

Supplementary Figure S9. Patient recruitment flowchart, showing the inclusion and exclusion process of the study cohort.

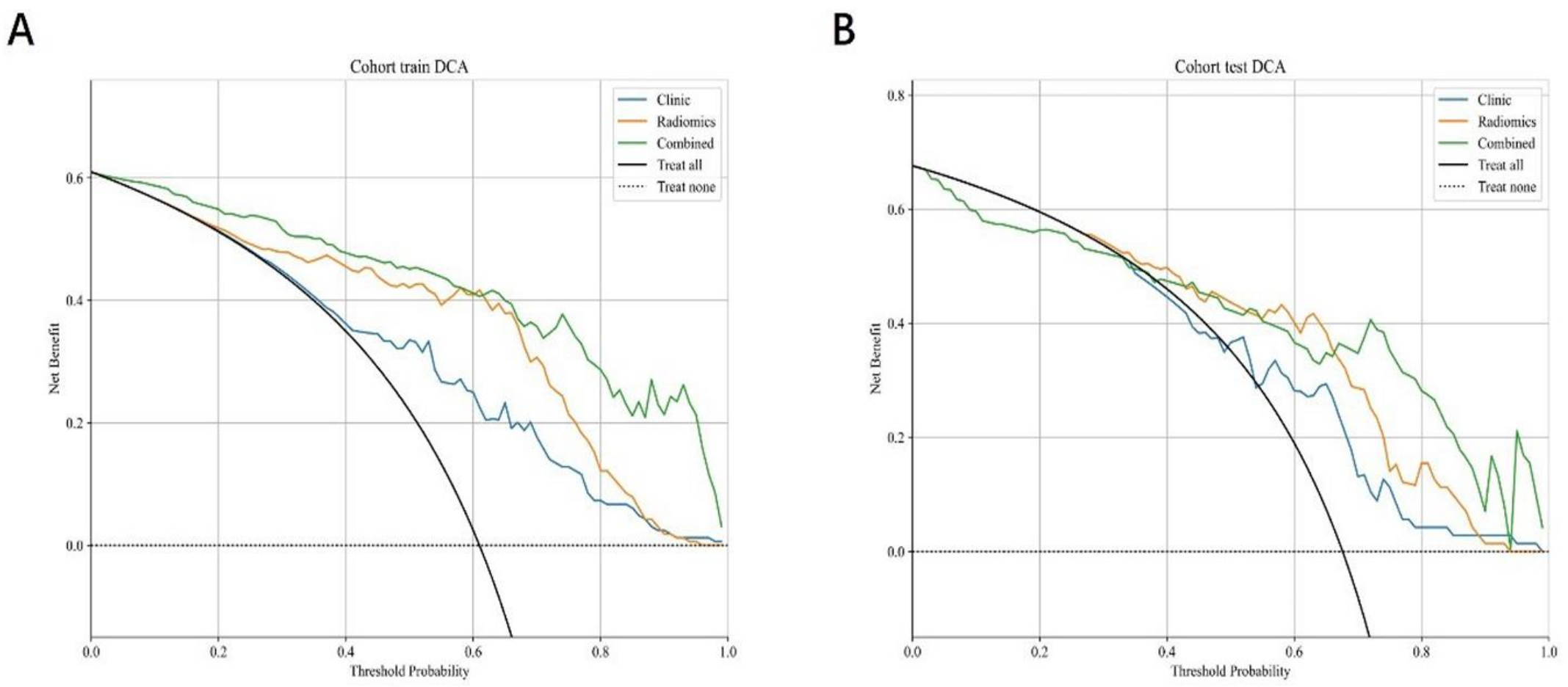

Supplementary Figure S10. Decision curve analysis (DCA) of the three models: (A) Training cohort; (B) Testing cohort.

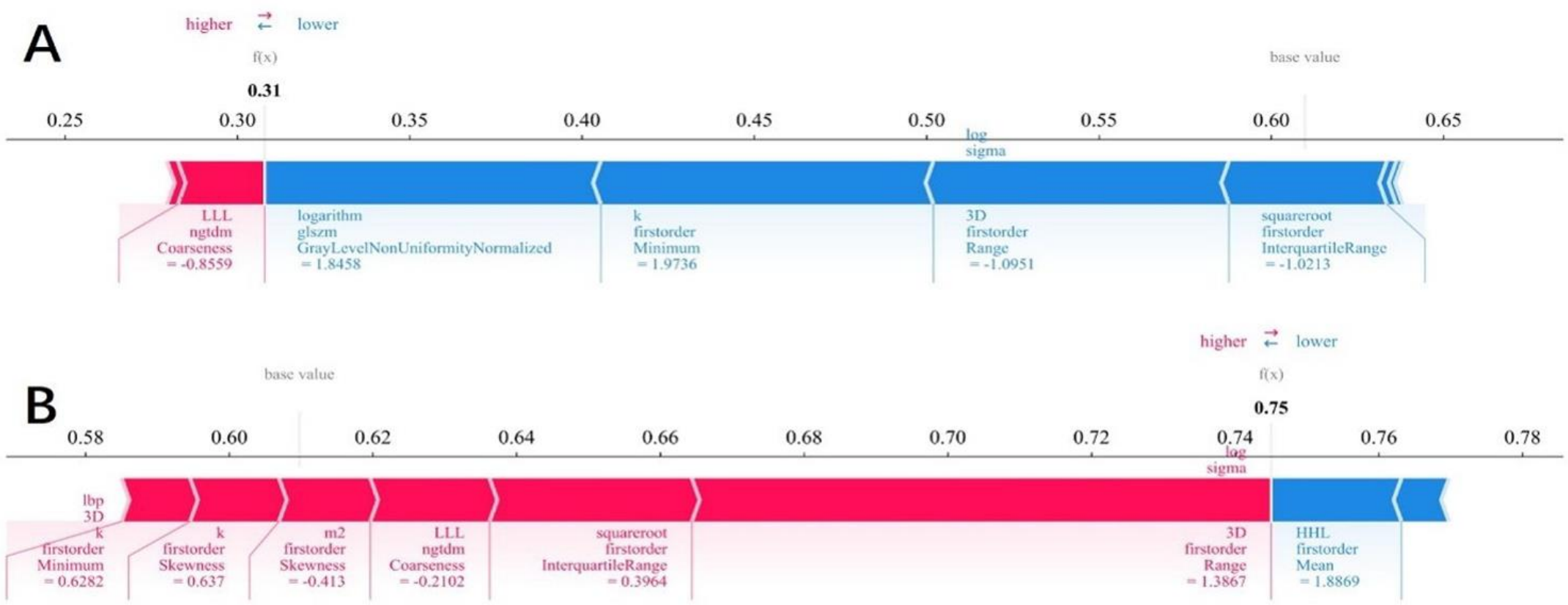

Supplementary Figure S11. SHAP force plots for representative cases: (A) Non-invasive pGGN case; (B) Invasive pGGN case, showing the direction and magnitude of each feature's contribution to individual predictions.
